# Bryostatin-1 improves function in arteries with suppressed endothelial cell autophagy

**DOI:** 10.1007/s11357-025-01650-5

**Published:** 2025-04-12

**Authors:** Jae Min Cho, Seul-Ki Park, Sohom Mookherjee, Emily Carolyn Peters, Paulo W. Pires, J. David Symons

**Affiliations:** 1 Department of Nutrition and Integrative Physiology, Division of Endocrinology, Metabolism and Diabetes, and Program in Molecular Medicine, University of Utah, Salt Lake City, UT USA; 2 Division of Cardiology, Department of Medicine, David Geffen School of Medicine, University of California, Los Angeles, CA USA; 3 Department of Medicine, VA Greater Los Angeles Health Care System, Los Angeles, CA USA; 4 Department of Physiology, University of Arizona, Tucson, AZ USA; 5 Sarver Heart Center, University of Arizona Health Science Center, Tucson, AZ USA

**Keywords:** Aging, Endothelial cell, Bryostatin-1, Nitric oxide

## Abstract

We have previously reported that when autophagy is suppressed in endothelial cells (ECs), a glycolytic defect limits shear-stress -induced ATP production to an extent that purinergic 2Y1 receptor (P2Y1R)-mediated activation of EC nitric oxide (NO) synthase (eNOS) is compromised. Subsequently we demonstrated the functional relevance of this finding in arteries from mice with genetic, pharmacological, and age-associated EC autophagy impairment. Using gain and loss of function approaches in vitro, we further revealed that p-PKCδ^T505^ serves as a signaling link between P2Y1R activation and NO generation. Here we sought to discern the functional relevance of this observation. First, shear-stress- induced activating phosphorylation of eNOS (p-eNOS^S1177^) that is otherwise prevented by knockdown of autophagy-related gene 3 (*Atg3*) in ECs was restored by the PKC agonist bryostatin-1. Next, in murine models of genetic and age-associated EC autophagy compromise, depressed vasodilation displayed by femoral and cerebral arteries was reversed by bryostatin-1 in a manner that could be prevented by concurrent NO synthase inhibition. Finally, the bryostatin-1-mediated normalization of intraluminal flow-induced vasodilation observed in femoral arteries from both models of EC autophagy disruption was mitigated by inhibiting downstream targets of p-PKCδ^T505^ i.e., p-PKD^S744/S748^ and p-PKD^S916^. These findings provide evidence that stimulating PKC/PKD has strategic potential to restore compromised endothelial function in pathologies associated with suppressed EC autophagy e.g., aging.

**Graphical Abstract:**

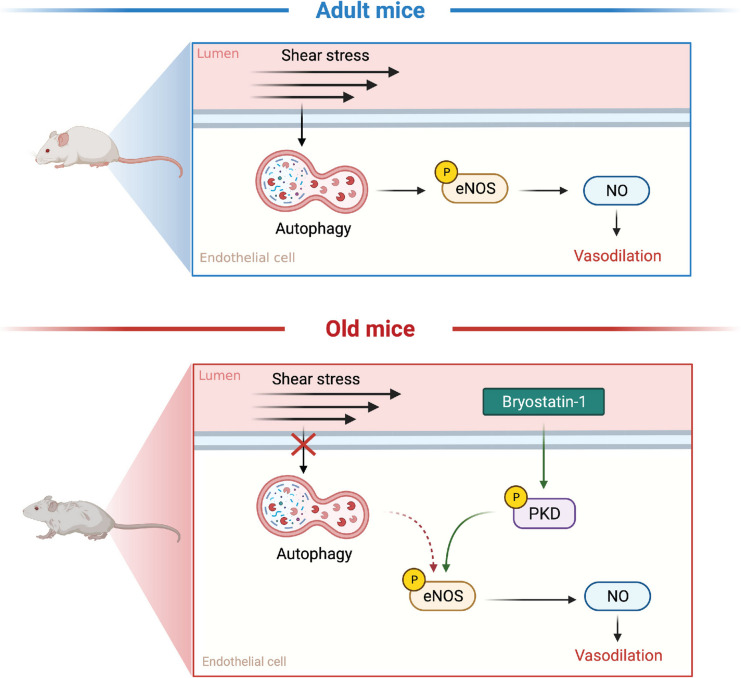

**Supplementary Information:**

The online version contains supplementary material available at 10.1007/s11357-025-01650-5.

## Introduction

As the proportion of the elderly in the U.S. population steadily increases, aging remains a major, unmodifiable risk factor for cardiovascular diseases, presenting significant individual and societal challenges [1]. Endothelial dysfunction is a hallmark of cardiovascular aging, and a decline in endothelial cell (EC) autophagy is increasingly recognized as a critical factor that contributes to vascular pathology [2, 3].

Earlier we revealed that repressed EC autophagy disrupts EC metabolism to an extent that attenuates EC function [4–6]. For instance, bovine (BAECs) and human (HAECs) arterial ECs upon genetic or pharmacological autophagy compromise [4, 5], and primary arterial ECs from older mice with impaired EC autophagy [6], exhibit reduced shear stress-induced glycolytic flux and ATP production. Consequences of this phenotype include diminished ATP/ADP-mediated purinergic 2Y1 receptor (P2Y1R) signaling via protein kinase Cδ (PKCδ) to endothelial nitric oxide (NO) synthase (eNOS), leading to reduced NO generation [5, 6].

After substantiating that genetic or pharmacological inhibition of P2Y1R signaling prevents shear-stress -induced NO generation in ECs with intact autophagy, whereas reactivating P2Y1R signaling with 2-Me-ADP restores shear-stress evoked NO generation in ECs with genetic repression of autophagy [5], we sought to determine the functional relevance of these in vitro findings. In this regard, we reported that 2-Me-ADP rejuvenates intraluminal flow-mediated vasodilation that is otherwise attenuated in femoral arteries from: (i) adult mice after pharmacological autophagy compromise; (ii) adult mice with inducible depletion of *Atg3* specifically in ECs; and (iii) older mice that display repressed arterial EC autophagy and EC glycolysis [6]. Results from that study led us to conclude that targeting purinergic signaling in contexts associated with repressed EC autophagy (e.g., aging) might be options to treat concurrent endothelial dysfunction.

The focus of the present investigation was to determine if pursuing another node in this pathway might yield functional benefits in settings associated with compromised EC autophagy. As mentioned, in earlier studies we deployed gain and loss of function approaches using cell-systems assays to reveal that p-PKCδ^T505^ serves as a signaling link between P2Y1R activation and NO generation. Here we sought to discern the translational relevance of these in vitro findings by testing the hypothesis that restoring PKCδ signaling improves intraluminal flow-mediated vasodilation that is otherwise compromised in rodent models of genetic and physiological EC autophagy compromise [7].

## Materials and methods

### Cell studies

#### Human aortic endothelial cells (HAECs)

HAECs (Lonza Inc, Bend, OR, USA) were maintained in endothelial basal medium 2 (EBM2; Lonza Inc, Bend, OR, USA) containing supplements (EGM- 2 SingleQuots, Lonza Inc) in a 5% CO_2_ atmosphere at 37 °C [5, 6, 8]. To delete the *Atg3* gene in HAECs we used CRISPR-Cas9 with single guide RNA targeting *Atg3* (sgAtg3) [6]. Briefly, the *Atg3* target sequence 5’-ACAAACGTGGCGAATAT- 3’ was designed and synthesized (Synthego, Menlo Park, CA, USA). Cas9 2 NLS (20 pM, Synthego) was combined with 60 pM synthetic sgRNA in 1X TE buffer and incubated for 10-min to allow the formation of the Cas9 RNP complex. Next, HAECs (1 × 10^5^ cells) were collected, rinsed with PBS, and the resultant pellet was suspended in 10 μl of buffer R (Thermo Fisher Scientific, Waltham, MA, USA). This suspension was combined in equal parts with the RNP complex, loaded into the Neon tip (Neon Transfection System; Thermo Fisher Scientific), and transfected (1400 V, 20 ms, 2 pulses).

#### Cell treatments

Three in vitro experiments were completed. First, we tested the hypothesis that *Atg3* knockdown prevents shear-stress-induced p-eNOS^S1177^ in a bryostatin-1 sensitive manner. HAECs transfected with sgRNA (sgAtg3) or non-targeting sgRNA (NC) were exposed to 0 (static) or 20 dyn/cm^2^ (shear-stress) laminar shear stress for 45-min following incubation with vehicle (PBS) or 50 nM bryostatin-1 (Tocris, Minneapolis, MN, USA) for 30-min. Next, we assessed the hypothesis that bryostatin-1 restores p-eNOS^S1177^ in HAECs by activating p-PKCδ^T505^ and its downstream target PKCµ (aka PKD) [9–13]. HAECs incubated for 45-min with vehicle (DMSO + ethanol), ii) 50 nM bryostatin-1, or iii) 50 nM bryostatin-1 + 10 µM rottlerin (Selleckchem, Houston, TX, USA). Finally, we tested if PKD inhibition using CRT0066101 (CRT) inhibits bryostatin-1 mediated p-eNOS^S1177^. HAECs were incubated for 45-min with i) vehicle, ii) bryostatin-1, or (iii) bryostatin-1 + 10 µM CRT (Cayman Chemical, Ann Arbor, MI, USA) for 45 min [14, 15].

#### Immunoblotting

Following the three in vitro experiments, cells were lysed in RIPA buffer containing protease and phosphatase inhibitors and EDTA [5, 6, 16–18]. Protein (25–35 μg) was resolved in 6X sample buffer and loaded into 4–20% SDS-PAGE gels (Bio-Rad, Hercules, CA, USA). Next, the proteins were transferred to a nitrocellulose membrane using a semi-dry transfer device (iBlot2, Thermo Fisher Scientific, Waltham, MA, USA). The membrane was blocked using 5% BSA for 1 h at room temperature and incubated with: (i) primary antibodies to LC3 (L8918, Sigma-Aldrich, St. Louis, MO, USA), p62 (ab91526, Abcam, Waltham, MA, USA), p-eNOS^S1177^ (#MA5-14957, ThermoFisher, Waltham, MA, USA), eNOS (#9572, Cell Signaling, Boston, MA, USA), GAPDH (#2118, Cell Signaling, Boston, MA, USA, p-PKD^S744/748^ (#2054, Cell Signaling, Boston, MA, USA), p-PKD^S916^ (#2051, Cell Signaling, Boston, MA, USA), and p-PKCδ^T505^ (#9374, Cell Signaling, Boston, MA, USA); followed by (ii) secondary antibodies to Goat anti-Rabbit and Goat anti-Mouse (for experiments involving PKD inhibition). Protein bands were detected by chemiluminescence and subsequently quantified using ImageJ (NIH, Bethesda, MD, USA).

#### Cell viability

To assess cell viability, HAECs were incubated with 10 μl PrestoBlue (Thermo Fisher Scientific, Waltham, MA, USA) for 30 min following the described treatments. Upon entering a living cell, PrestoBlue is reduced from resazurin, a blue compound that lacks fluorescence, to resorufin, a red compound that is highly fluorescent. Fluorescence intensity, that is proportional to metabolically active cells, was measured using a microplate reader set to an excitation wavelength of 560 nm and an emission wavelength of 590 nm [5, 6]. To measure apoptosis, HAECs incubated with Alexa Fluor 488 Annexin V and propidium iodide (PI) following the described treatments, using guidelines provided in the FITC Annexin V/Dead Cell Apoptosis Kit (Thermo Fisher Scientific, Waltham, MA, USA). Phosphatidylserine shifts externally on the cell membrane during the process of apoptosis, binding to FITC-tagged Annexin V, and fluoresces green. Dead cells fluoresce red and green, whereas live cells show little to no fluorescence. Using flow cytometry (Becton Dickinson), cells were categorized as early apoptotic, late apoptotic, dead, or viable [5, 6].

### Animal studies

#### Wild type and Atg3^EC−/−^ mice

*Atg3*^*flox/flox*^ mice [5, 6] and Cdh5-CreERT2 animals [19] on a C57BL/6 background were bred to produce *Atg3*^*flox/flox*^/Cdh5-CreERT2 (*Atg3*^*EC−/−*^) (27 ± 1 g) and *Atg3*^*flox/flox*^ littermates (WT) (27 ± 1 g). Floxed *Atg3* and *Cdh5*-Cre was confirmed by PCR analysis of genomic DNA. To activate Cdh5-Cre in 6-month old animals, 4 mg tamoxifen was administered via oral gavage to all mice for 4 consecutive days. Femoral and cerebral arterial segments were obtained from WT and *Atg3*^*EC−/−*^ mice two weeks following the last tamoxifen administration [6]. At the time of study, mice were anesthetized using 2–5% inhaled isoflurane (1-chloro-2,2,2-trifluoroethyl difluoromethyl ether) combined with 100% oxygen. When a stable plane of anesthesia was attained, the chest was opened, the heart exposed, and the left ventricle was perfused with 3 ml PBS after making a small incision in the right atrium. Next, the heart was excised, the brain was removed, and arteries (femoral, cerebral, parenchymal) were isolated from adherent tissue while bathed in iced PBS, and segments were placed in myograph chambers to assess vasomotion ex vivo using isobaric procedures [6, 20–23].

#### Adult and old mice

Male C57BL/6 mice were obtained from: (i) Jackson Laboratories (C57BL/6; Bar Harbor, ME) at 5–6 months of age, and used for experiments at 7 months of age (adult) (31 ± 1 g); or (ii) the National Institute on Aging (C57BL/6) rodent colony at 18 months of age, and studied at 24 months of age (old) (33 ± 1 g) [6, 17]. Between the time of mouse delivery and data collection, animals were housed at the University of Utah in AALAC accredited facilities, 4 per cage, and maintained on a 12:12 h light: dark cycle in a temperature-controlled environment (22–23°C). Standard rodent chow and water were provided ad libitum. Vessels were obtained using procedures described earlier.

#### Vascular reactivity: femoral arteries

After their isolation, femoral artery segments were immersed in 4 °C physiological saline solution (PSS) containing (mM): 145.0 NaCl, 4.7 KCl, 2.0 CaCl_2_, 1.17 MgSO_4_, 5.0 glucose, 2.0 pyruvate, 0.02 EDTA, 3.0 MOPS buffer, 10 g/L of BSA at pH 7.4. Each end of the vessel was cannulated using a micropipette tip with the aid of a dissecting microscope (SZX10; Olympus). After the temperature of the bathing medium was increased over 30 min to 37°C, arteries were equilibrated for one hour, followed by 10 mmHg increases in intraluminal pressure every 5 min to 60 mmHg. Five responses were observed in one femoral artery per mouse. First, a cumulative concentration–response curve to potassium chloride (KCl, 20-100 mM) was obtained to measure non-receptor-mediated vasoconstriction. Second, to measure receptor-mediated vasoconstriction, a cumulative concentration–response curve to phenylephrine (PE, 10^–8^−10^–5^ M) was obtained, and the dose of PE required to evoke 50% of maximal PE-evoked constriction was calculated (see below). Third, after intraluminal incubation with the respective vehicle, and upon 50% of maximal PE-induced vasoconstriction, the intraluminal flow was initiated by increasing the inflow pressure (e.g., P1) and decreasing the outflow pressure (e.g., P2) to create a gradient (e.g., ΔP) across the vessel. For example, from isobaric conditions wherein P1 = 60 mmHg = P2, a ΔP of 6 mmHg was created by increasing P1 to 63 mmHg and decreasing P2 to 57 mmHg. Percent vasodilation was measured in response to ΔP’s of 6, 12, 18, and 24 mmHg × 3 min each [6]. Fourth, after arteries equilibrated in an isobaric environment (60 mmHg) for 30-min in the presence of intraluminal: (i) NOS inhibition using N^G^-Methyl-L-arginine acetate salt (L-NMMA) (1 mM μM); (ii) bryostatin-1 (50 nM); (iii) bryostatin-1 and L-NMMA (1 mM); or (iv) bryostatin-1 and CRT (5 µM), a second intraluminal flow-mediated vasodilation curve was completed after stable PE-induced preconstruction. Fifth, after arteries equilibrated in an isobaric environment (60 mmHg) for 30 min and upon PE-induced preconstriction, a concentration–response curve to the endothelium-independent vasodilator sodium nitroprusside (SNP, 10^–9^—10^–4^ M) was completed to estimate vascular smooth muscle function. Femoral artery vascular responses were evaluated in WT and *Atg3*^*EC−/−*^ mice and adult and older mice.

#### Vascular reactivity: middle cerebral (MCA) and anterior cerebral arteries (ACA)

While the brain was bathed in 4°C PSS, segments of MCA and ACA were isolated from the Circle of Willis. Next, each end of the vessel was cannulated using a micropipette tip while immersed in a myograph chamber, and the temperature of the bathing medium was increased over 30-min to 37°C, followed by 10 mmHg increases in intraluminal pressure every 5 min to 60 mmHg. First, the MCA/ACA were equilibrated in an isobaric environment (60 mmHg) for 30 min. Second, after a 30-min washout period, 3 µM phenylephrine (PE) was used to preconstrict the artery, and vasodilation was measured in response to 10^–2^ M acetylcholine (ACh). Third, upon washout of the drugs and 30-min incubation of the MCA/ACA with 50 nM bryostatin-1, PE-induced precontraction was initiated followed by responses to 10^–2^ M ACh. Thirty-min later (fourth), after exchanging the vessel bathing medium with PSS, responses to 10^–2^ M SNP were evaluated in arteries precontracted with 3 µM PE. For MCA, ACA, and femoral arteries, percent vasodilation was calculated as (DT-Dp)/(Di-Dp) × 100. Where DT is the recorded diameter at a given time point (e.g., diameter response to flow or SNP), Dp is the diameter recorded after the addition of the vasoactive agent (i.e. pre-constriction diameter), and Di is the diameter recorded immediately before the addition of the vasoactive agent (initial diameter). Percent vasoconstriction (% of baseline) was calculated as Dp/Di × 100 [6, 20]. MCA and ACA responses were evaluated in WT and *Atg3*^*EC−/−*^ mice, and adult and older mice.

#### Vascular reactivity: parenchymal arterioles

After their isolation, parenchymal arterioles were transferred to a myograph chamber. One end of the vessel was cannulated onto a glass cannula (~ 30 µm inner diameter at the tip) and filled with PSS. The other end was tied to the cannula forming a blind sac [24]. Next, the cannula was filled with artificial cerebrospinal fluid (aCSF in mM: 124 NaCl, 3 KCl, 2 MgCl_2_, 1.085 NaH_2_PO_4_•2H_2_0, 26 NaHCO_3_, 2.0 CaCl_2_, 4 Dextrose, pH 7.4). The pressure myography chamber was then transferred to an inverted microscope and connected to a gravity-dependent column of water for pressurization. Preparations were superfused with warm (37ºC), oxygenated (21% O_2_/5%, CO_2_/74% N_2_) aCSF exchanged at a rate of 3–5 mL per minute for approximately 30 min at a pressure of 15 mmHg to equilibrate. Intraluminal pressure was then increased and maintained to 40 mmHg for generation of spontaneous myogenic tone. Outer and lumen diameters were constantly recorded in real-time at 15 Hz using the IonWizard v7.3 software (IonOptix, Westwood, MA). Spontaneous myogenic tone was calculated as: Myogenic tone (%) = [1- (LD_T_/LD_P_)]* 100 [25], where LD_T_ is the lumen diameter at stable spontaneous myogenic tone and LD_P_ is the passive lumen diameter. Preparations that did not reach at least 15% myogenic tone were considered non-viable and excluded from further analysis.

Following generation of spontaneous myogenic tone, preparations were exposed to i) vehicle (PSS) or ii) bryostatin- 1 (50 nM) after incubation for 10 min with 1 μM 2-Me-ADP in the superfusate. At the end of the experiment, aCSF containing test agent was replaced by fresh aCSF and arterioles were allowed to return to basal myogenic tone. Viability of the preparation was assessed after exposure to 60 mM KCl aCSF (balanced NaCl). Passive lumen diameter was recorded under Ca^2+^-free aCSF supplemented with 2 mM EGTA + 10 µM diltiazem (L-type voltage-gated Ca^2+^ channel blocker) + SNP (10 µM). Dilation data are shown as Vasodilation (%), calculated: [(LD_D_ – LD_B_)/LD_P_) × 100], where LD_D_ is the lumen diameter at peak dilation; LD_B_ is lumen diameter at baseline (immediately before addition of drug); LD_P_ is the passive lumen diameter [26].

#### Statistical analyses

Data are presented as mean ± standard error of the mean (SEM). Statistical analyses were performed by GraphPad Prism software version 9 (Boston, MA, USA). For comparisons involving two factors, a two-way ANOVA was conducted. Comparisons among three groups were analyzed by one-way ANOVA. If significant differences existed among groups, Tukey’s multiple comparisons test was used to identify the location of the differences. For comparison between two groups, an unpaired t-test was performed. Two-way repeated measures ANOVA was used to determine significance concerning intraluminal flow-mediated vasodilation in arteries. Note that information concerning statistical tests are included in each figure and table legend. Statistical significance represents *p < 0.05, **p < 0.01.

## Results

### Bryostatin-1 restores p-eNOS^S1177^ in HAECs upon Atg3 knockdown

Earlier we showed that physiologically-relevant shear stress [4–6, 27] increases glycolytic ATP production leading to P2Y1R-mediated p-eNOS^S1177^ activation via PKCδ^T505^ in BAECs transfected with scrambled but not *Atg3* siRNA [4, 5]. Supporting a central role for PKC in relaying the autophagy-dependent purinergic-mediated signal to eNOS in BAECs, results from additional experiments revealed that shear-induced p-eNOS^S1177^ is restored in autophagy-impaired BAECs via pharmacological (e.g., bryostatin-1) or genetic (e.g., CA-PKCδ) activation of PKCδ^T505^ [5]. Here we sought to confirm the main findings in HAECs by testing the hypothesis that bryostatin-1 restores shear-stress -induced p-eNOS^S1177^ that is otherwise repressed upon Atg3 compromise.

**Fig. 1:**
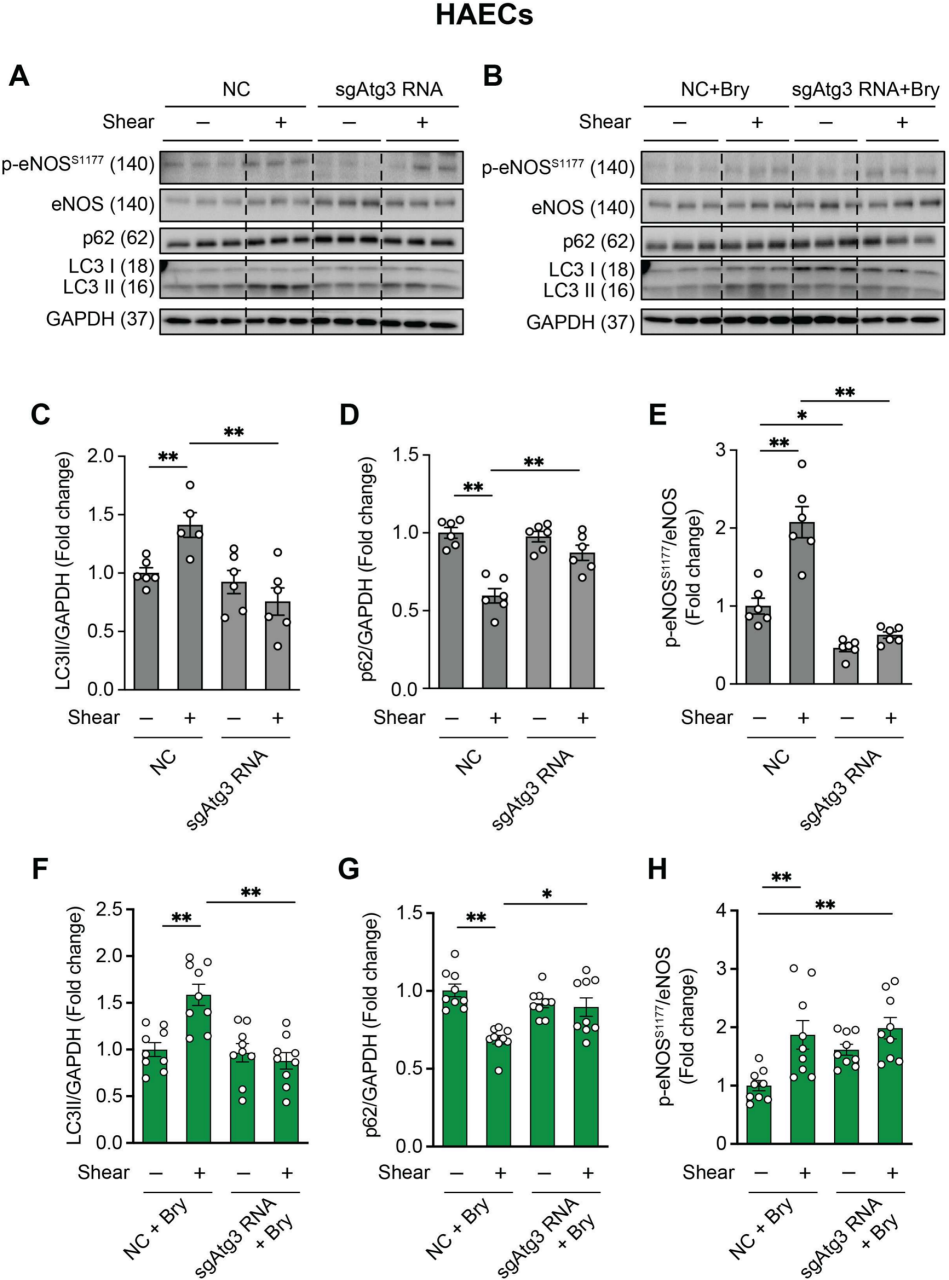
Shear stress-induced p-eNOS^S1177^ in HAECs is prevented by Atg3 knockdown but is restored by bryostatin-1. HAECs were transfected with non-targeting (NC) or single-guide Atg3 RNA (sgRNA) using CRISPR-Cas9. HAECs treated with vehicle (PBS) or 50 nM bryostatin-1 (Bry) were exposed to 0 (- shear) or 20 dyne • cm^2^ shear stress (+ shear) for 45 min. Representative images **(A, B)** and mean densitometry of LC3II/GAPDH **(C, F)**, p62/GAPDH **(D, G)**, and p-eNOS^S1177^/eNOS **(E, H)** protein expression are shown. For C-H, *p < 0.05, **p < 0.01. *p* values were obtained using two-way ANOVA. Data are presented as mean ± STE, with symbols within each histogram representing one well of a 6-well plate

Atg3 mediates lipidation of Atg8/LC3I to form mature LC3 (microtubule-associated protein light chain 3) -II and is required for the formation of mature autophagosomes [28]. The adaptor protein p62 (A170/sequestosome-1) tethers/escorts damaged/dysregulated organelles inside the autophagosome and is degraded upon fusion with the lysosome [29–31]. As would be predicted based on our previous findings, shear-stress increased LC3-II:GAPDH accumulation, p62 degradation, and p-eNOS^S1177^:eNOS activation, in HAECs transfected with non-targeting but not Atg3 sgRNA (Fig. 1A and 1 C-E). Importantly, treating HAECs upon Atg3 sgRNA with bryostatin-1 normalized shear-induced p-eNOS^S1177^:eNOS without impacting LC3-II:GAPDH or p62:GAPDH (Fig. 1B and 1 F-H). Bryostatin-1 treatments did not influence cell death or apoptosis (Fig S1).

### Bryostatin-1 improves arterial function in Atg3^EC−/−^ mice

**Fig. 2:**
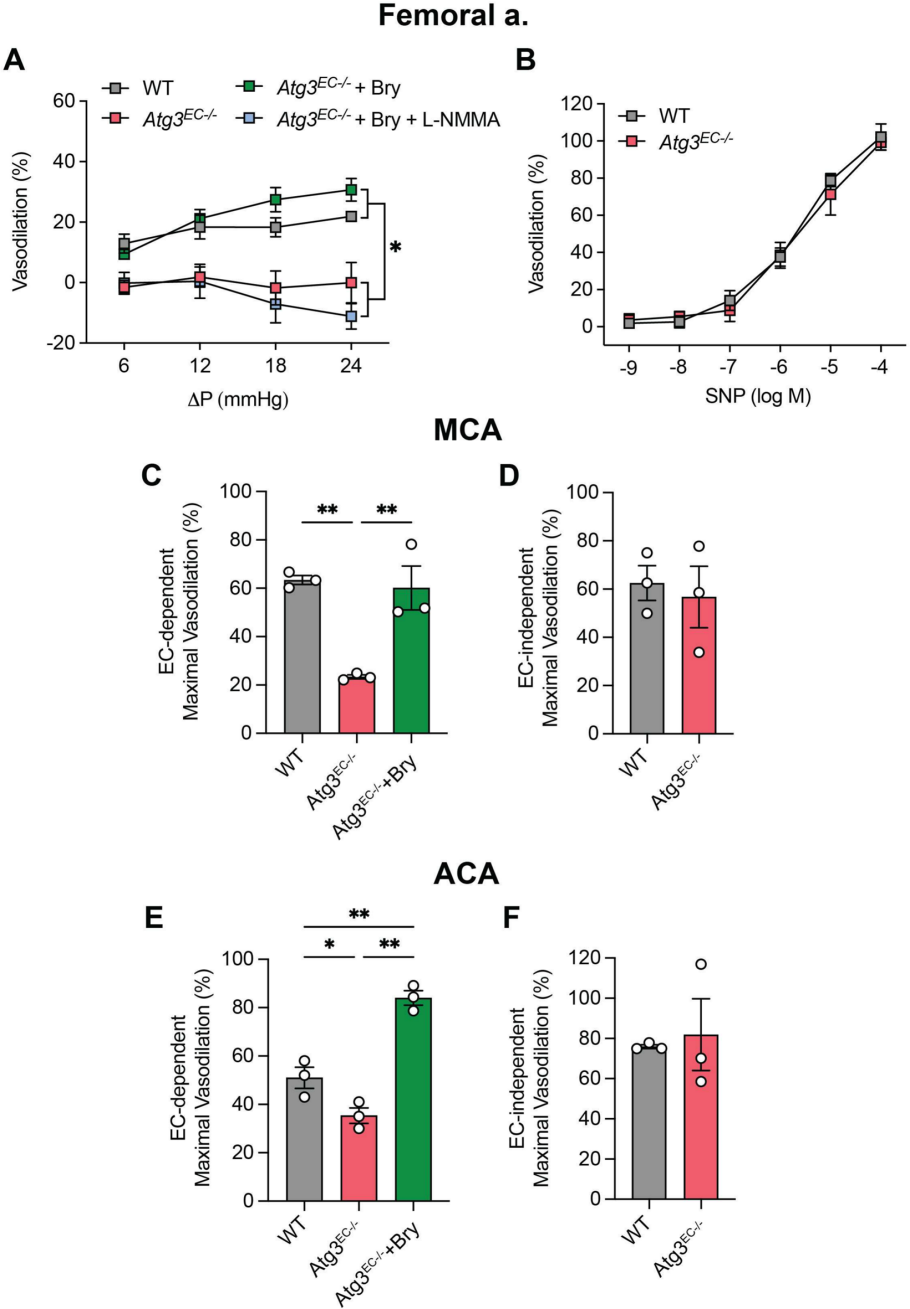
Endothelium-dependent vasodilation is compromised in femoral and cerebral arteries from *Atg3*^*EC−/−*^ vs. WT mice but is restored by bryostatin-1. Compared to intraluminal flow-mediated vasodilation in femoral arteries from WT mice, responses were compromised in vessels from *Atg3*^*EC−/−*^ mice, but could be restored upon incubation with 50 nM bryostatin-1 (Bry). Bry-induced improvements were prevented by concurrent treatment with 10^–3^ M L-NMMA **(A)**. Vascular smooth muscle responses to sodium nitroprusside (SNP) were similar between WT and *Atg3*^*EC−/−*^ mice **(B)**. For A, *p < 0.05 vs. WT. For A, B, significance was assessed via two-way repeated measures ANOVA. Each symbol represents the mean value obtained from 4 mice per group, 1 vessel segment per mouse. Acetylcholine-evoked vasodilation was impaired in middle (MCA, **C**) and anterior (ACA, **E**) cerebral arteries from *Atg3*^*EC−/−*^ vs. WT mice, but could be restored by concurrent incubation with 50 nM Bry. Vascular smooth muscle responses to SNP were similar between WT and *Atg3*^*EC−/−*^ mice **(D, F)**. For C and E, *p < 0.05, **p < 0.01. *p* values were obtained using a one-way ANOVA, followed by a Tukey post-hoc test when appropriate. For D, F, significance was assessed using an unpaired t-test. For C-F, symbols within histograms represent maximal vasodilation to 10^–2^ M acetylcholine (C, E) or 10^–4^ M SNP (D, F) from 3 mice, 1 vessel segment per mouse

In vitro findings that bryostatin-1 restored shear-induced p-eNOS^S1177^ in the context of genetic EC autophagy compromise inspired us to study functional relevance ex vivo using femoral and cerebral arteries from adult mice with tamoxifen-inducible depletion of *Atg3* specifically in ECs [6]. Substantiating results we reported earlier [6], intraluminal flow-mediated vasodilation was blunted in femoral arteries from *Atg3*^*EC−/−*^ vs. WT mice (Fig. 2A). In support of the in vitro data, treating femoral arteries with bryostatin- 1 restored intraluminal flow-mediated vasodilation in vessels from *Atg3*^*EC−/−*^ mice. Indicating an important contribution to vasodilation from NO in this context, bryostatin-1 -evoked improvements in femoral arteries from *Atg3*^*EC−/−*^ mice were prevented by co-incubation with L-NMMA (Fig. 2A). Endothelium-independent vasodilation (Fig. 2B), and non-receptor (Fig S2**A**) and receptor (Fig S2**B**) -mediated vasocontriction, was similar in femoral arteries from *Atg3*^*EC−/−*^ and WT mice. Regarding cerebral arteries, bryostatin-1 improved acetylcholine-evoked vasodilation that was otherwise depressed in MCA (Fig. 2C) and ACA (Fig. 2E) from *Atg3*^*EC−/−*^ vs. WT mice. However, endothelium-independent vasodilation was similar in MCA (Fig. 2D) and ACA from both groups (Fig. 2F). These results, coupled with our in vitro data described earlier, strongly suggest bryostatin- 1 reactivates p-eNOS^S1177^ in the setting of genetic EC autophagy compromise to an extent that improves arterial function. Vessel characteristics are shown in Supplementary Table 1.

### Bryostatin-1 improves arterial function in older mice

**Fig. 3:**
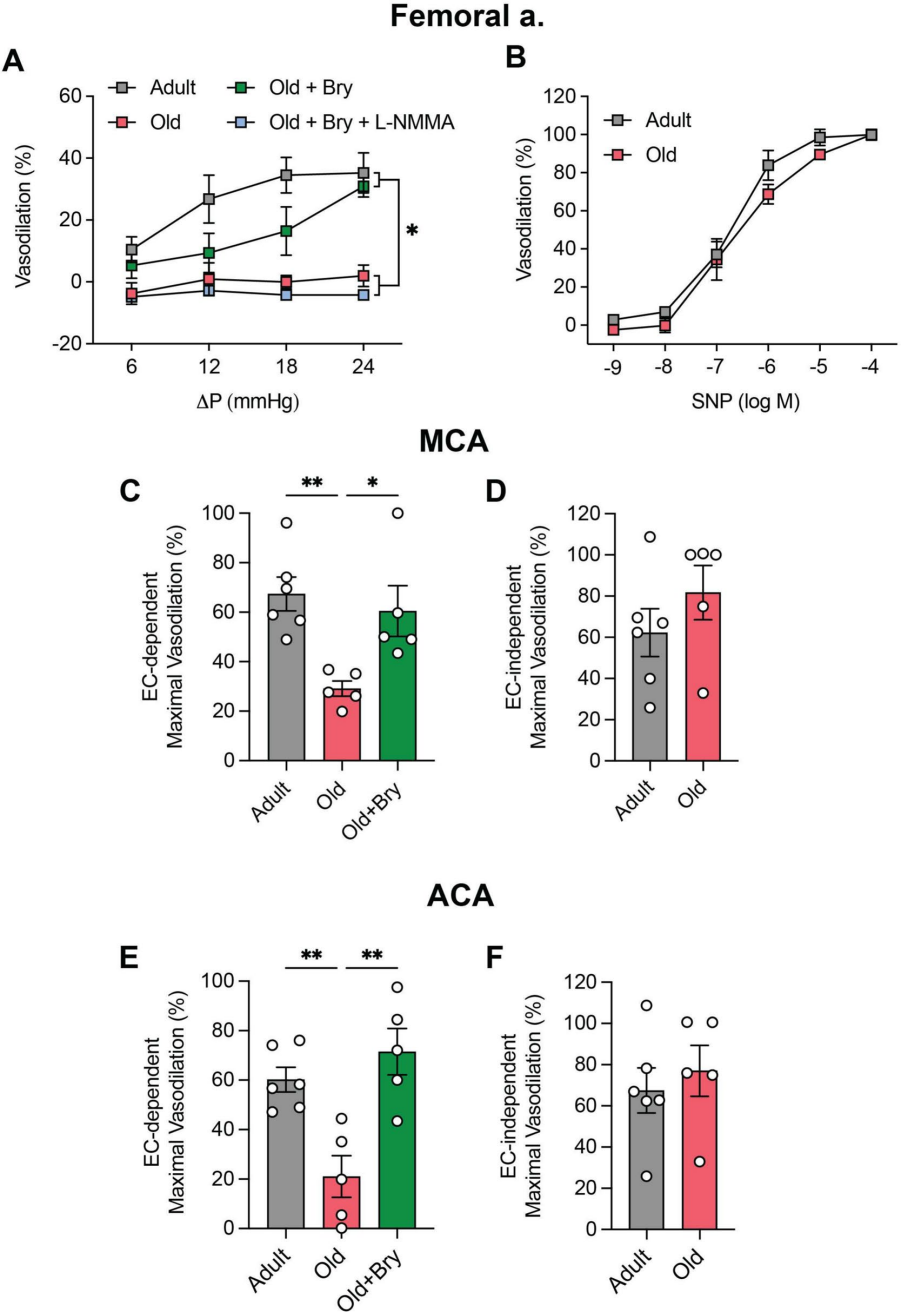
Endothelium-dependent vasodilation is compromised in femoral and cerebral arteries from old vs. adult mice, but is restored by bryostatin-1. Compared to intraluminal flow-mediated vasodilation in femoral arteries from adult mice, responses were compromised in vessels from older animals, but could be restored upon incubation with 50 nM bryostatin-1 (Bry). Bry-induced improvements were prevented by concurrent treatment with 10^–3^ M L-NMMA **(A)**. Vascular smooth muscle responses to sodium nitroprusside (SNP) were similar between groups **(B)**. For A, *p < 0.05 vs. adult. For A, B, significance was assessed via two-way repeated measures ANOVA. Each symbol represents the mean value obtained from 5–6 mice, 1 vessel segment per mouse. Acetylcholine-evoked vasodilation was impaired in middle (MCA, **C**) and anterior (ACA, **E**) cerebral arteries from old vs. adult mice, but could be restored by concurrent incubation with 50 nM Bry. Vascular smooth muscle responses to SNP were similar between groups **(D, F)**. For C-E, *p < 0.05, **p < 0.01. *p* values were obtained using a one-way ANOVA, followed by a Tukey post-hoc test when appropriate. For D, F, significance was assessed using an unpaired t-test. For C-F, symbols within histograms represent maximal vasodilation to 10^–2^ M acetylcholine (C, E) or 10^–4^ M SNP (D, F) from 5 mice, 1 vessel segment per mouse

To complement findings observed in arteries from mice with genetic depletion of EC autophagy, we deployed a model of physiological EC autophagy repression i.e., aging. In this regard, we reported that basal autophagy, together with shear-induced autophagic flux, glycolysis, ATP production, p-eNOS^S1177^, and NO generation are lower in primary arterial ECs from 24-month vs. 7-month-old C57BL/6 mice [6]. Here, intraluminal flow-mediated vasodilation was impaired in femoral arteries from older vs. adult mice in a manner that was recapitulated in arteries from adult mice by concurrent treatment with L-NMMA. Of note, bryostatin-1 normalized flow-mediated vasodilation in arteries from older mice in an L-NMMA-sensitive manner (Fig. 3A). Endothelium-independent function (Fig. 3B), together with non-receptor (Fig S2**C**) and receptor-mediated (Fig S2**D**) vasoconstriction, was similar between adult and older mice. Regarding cerebral arteries, bryostatin-1 improved acetylcholine-evoked vasodilation that was otherwise depressed in MCA (Fig. 3C) and ACA (Fig. 3E) from older vs. adult mice. Substantiating our earlier findings, vascular smooth muscle function was similar in MCAs (Fig. 3D) and ACAs (Fig. 3F) from both groups.

### Bryostatin-1-mediated p-eNOS^S1177^ activation is sensitive to PKC inhibition

**Fig. 4:**
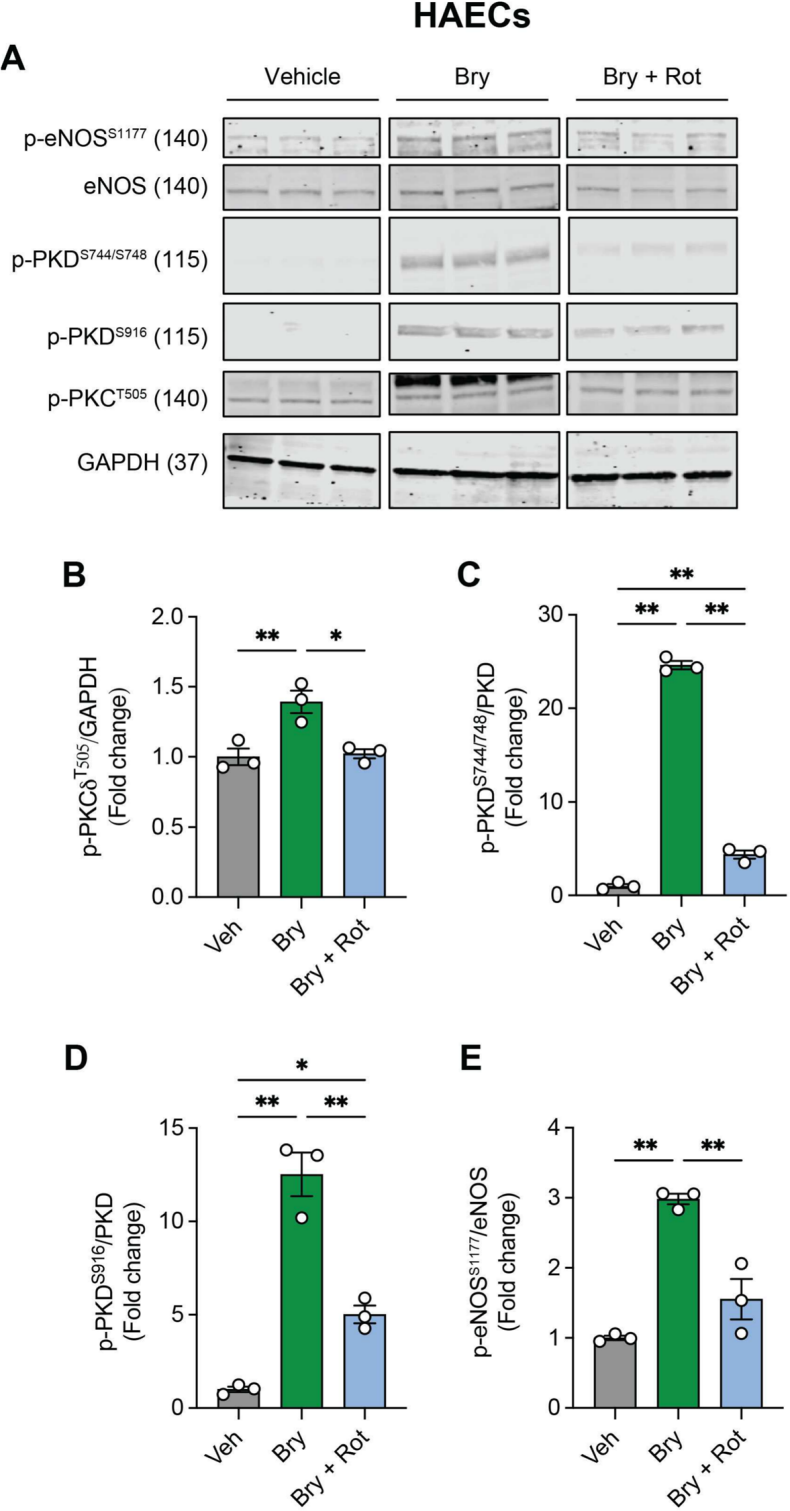
Bryostatin-1-induced p-PKCδ^T505^, p-PKD^S744/748^, p-PKD^S916^, and p-eNOS^S1177^ in HAECs is attenuated by rottlerin. HAECS were treated with vehicle (Veh, DMSO + ethanol), 50 nM bryostatin-1 (Bry), or Bry + 10 μM Rottlerin (Bry + Rot). Representative images **(A)** and mean densitometry (**B-D**) are shown. Bry-induced increases in p-PKCδ^T505^, p-PKD^S744/748^, p-PKD^S916^, and p-eNOS^S1177^ were attenuated by concurrent treatment with Rot. For B-E, *p < 0.05, **p < 0.01. *p* values were obtained using a one-way ANOVA, followed by a Tukey post-hoc test when appropriate. Symbols within each histogram represent one 100 mm dish.

To this point we observed that bryostatin-1 restores shear-induced p-eNOS^S1177^ that is otherwise prevented by EC autophagy compromise (Fig. 1), and that this finding has functional relevance because bryostatin-1 improves endothelial function in models of genetic (Fig. 2) and physiological (Fig. 3) EC autophagy disruption. Based on our previous report [5, 6], we hypothesized that bryostatin-1 stimulates p-eNOS^S1177^ in ECs by activating p-PKCδ^T505^. After determining an efficacious rottlerin treatment regimen (i.e., 10 μM for 45 min) in HAECs (**Fig S3**), we observed bryostatin-1 heightens p-PKCδ^T505^ and its downstream targets p-PKD^S744/S748^, p-PKD^S916^, and p-eNOS^S1177^ in a manner that can be abrogated by p-PKCδ^T505^ inhibition using 10 μM of rottlerin (Fig. 4** A-E**).

### Bryostatin-1-mediated improvements in arterial function are sensitive to PKD inhibition

**Fig. 5:**
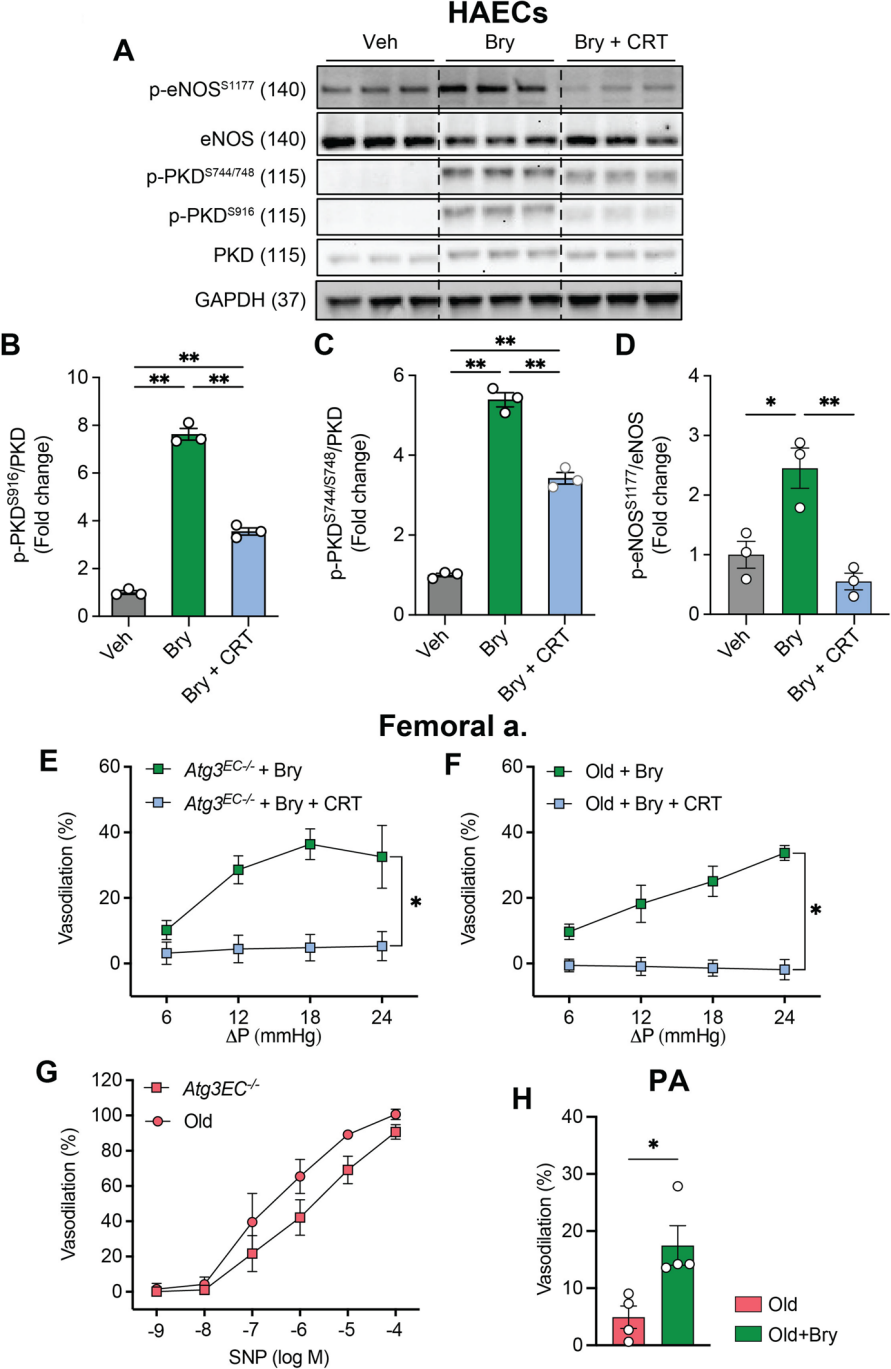
Bryostatin-1-induced p-PKD^S744/748^, p-PKD^S916^, p-eNOS^S1177^, and intraluminal flow-mediated vasodilation is attenuated by CRT0066101. HAECs were treated with vehicle (Veh), 50 nM bryostatin-1 (Bry), or Bry + 5µM CRT0066101 (Bry + CRT). Representative images **(A)** and mean densitometry (**B-D**) are shown. Bry-induced increases in p-PKD^S744/748^, p-PKD^S916^, and p-eNOS^S1177^ were attenuated by concurrent treatment with CRT. For B-D, *p < 0.05, **p < 0.01. *p* values were obtained using a one-way ANOVA, followed by a Tukey post-hoc test when appropriate. Symbols within each histogram represent one well of a 6-well plate. Concurrent treatment with CRT prevented intraluminal flow-mediated vasodilation that was otherwise intact in femoral arteries from *Atg3*^*EC−/−*^ mice treated with Bry **(E)**. Likewise, CRT abolished intraluminal flow-mediated vasodilation observed in femoral arteries from older mice treated with Bry **(F)**. Vascular smooth muscle responses were intact in both groups **(G)**. Relative to parenchymal arterioles from older mice treated with vehicle, Bry increased vasodilation **(H)**. For E, F significance was assessed via two-way repeated measures ANOVA. Each symbol represents the mean value obtained from 4–6 mice, 1 vessel segment per mouse. For E, F *p < 0.05 between groups at each flow rate. For H, significance was assessed via an unpaired t-test. Each symbol within the histogram represents a parenchymal arteriolar segment

Because we observed bryostatin-1-induced PKD activation (i.e., p-PKD^S744/S748^, p-PKD^S916^) to be markedly more robust than PKCδ^T505^ upregulation (Fig. 5** A-D**), and since we are aware of reports that rottlerin exerts off-target effects in other cell types [5], we reasoned that PKD inhibition might be the best approach to evaluate functional consequences of manipulating this node in the pathway [14, 15]. First we investigated the efficacy of CRT to inhibit PKD activation using a cell-systems approach. In HAECs, bryostatin-1-induced p-PKD^S744/S748^, p-PKD^S916^, and p-eNOS^S1177^ were abrogated by concurrent CRT treatment (Fig. 5A-D), in the absence of cell death or apoptosis (**Fig S4**). Based on these results, intraluminal flow-mediated vasodilation experiments were completed in the absence and presence of CRT to evaluate functional consequences of PKD inhibition. Our findings indicate that intraluminal flow-mediated vasodilation in the presence of bryostatin-1 is prevented in arteries from mice with genetic (Fig. 5E) or aging-associated (Fig. 5F) EC autophagy compromise by concurrent incubation with the PKD inhibitor CRT, and that vascular smooth muscle function is intact in both models (Fig. 5G). As important proof of concept for future investigations, we show that bryostatin-1 improves parenchymal arteriolar vasodilation in older mice (Fig. 5H). Collectively, our data indicate bryostatin-1 activates PKC/PKD signaling to improve arterial function in mice with repressed EC autophagy.

## Discussion

Aging is a major and unmodifiable risk factor for cardiovascular disease [2, 3]. The number of persons ≥ 65 years of age will comprise ~ 20% of the U.S. population by 2030 [1], creating a challenge for health-provider systems in general, and individual care-givers in particular. Direct and indirect costs associated with aging-related cardiovascular pathologies are escalating and collectively pose an enormous societal challenge [32]. While research efforts are robust concerning mechanisms responsible for aging-evoked endothelial dysfunction and advancements in this regard are steady, precise targets for therapeutic intervention remain elusive.

### Repression of EC autophagy associates with cardiovascular pathologies

Studies by others [33, 34] and ourselves[6] suggest that age-driven declines in EC autophagy associate strongly with vascular dysfunction, and we identified a novel mechanistic link between repressed EC autophagy and compromised EC NO production and arterial vasodilation. Specifically, we found that disrupting EC autophagy reduces glycolytic flux and ATP production, leading to a concomitant decline in autocrine signaling via the P2Y1R to eNOS [4–6]. Of note, we could restore signaling to eNOS in autophagy-deficient ECs by genetically amplifying GLUT1 expression, or pharmacological (via 2-Me-ADP or exogenous ATP/ADP) activation of P2Y1Rs [6].

Supporting the idea that this novel pathway has relevance to the vascular dysfunction that accompanies aging, primary arterial ECs from aged mice display reduced shear-stress -evoked autophagic flux, GLUT1 expression, glycolytic ATP production, eNOS activation, and NO generation in vitro [6]. Importantly, these changes occurred in concert with defective intraluminal, NO-dependent, flow-mediated vasodilation in arteries from aged mice examined ex vivo [6]. Demonstrating a functional relevance of the pathway revealed in these cell-systems assays, 2-Me-ADP rejuvenates intraluminal flow-mediated vasodilation that is otherwise attenuated in femoral arteries upon pharmacological (e.g., 3-MA), genetic (e.g., *Atg3*^*EC−/−*^ mice), or physiological (e.g., 24-mo mice) autophagy disruption [6]. Results from that study led us to conclude that targeting purinergic signaling in contexts associated with repressed EC autophagy (e.g., aging) might be options to treat concurrent endothelial dysfunction.

### PKC/PKD signaling links purinergic signaling to eNOS activation

In this report we sought to determine whether targeting the link between P2Y1R stimulation and eNOS activation also has functional relevance. As background, da Silva et al. first reported extracellular nucleotide-mediated activation of p-eNOS^S1177^ in human umbilical vein ECs exposed to shear stress occurs via p-PKCδ^T505^ [35]. We confirmed this observation in arterial ECs, and further revealed that shear-induced p-PKCδ^T505^, p-eNOS^S1177^ and NO generation are prevented in ECs transfected with *Atg3* siRNA or PKCδ siRNA vs. the appropriate scrambled siRNA, and in BAECs with intact autophagy treated with the PKCδ inhibitor rottlerin [5]. Using a gain-of-function approach, when BAECs with suppressed autophagy were transfected with constitutively active PKCδ and subsequently challenged with shear stress, p-eNOS^S1177^ and NO generation were restored, and indices of autophagy were not altered [5]. Finally, confirming that p-PKCδ^T505^ is a downstream target of purinergic-mediated signaling, we observed this kinase to be refractory to shear stress in BAECs with intact autophagy after transfection with P2Y1R vs. scrambled siRNA. Employing a complementary approach to substantiate these data, shear stress evoked robust p-PKCδ^T505^, p-eNOS^S1177^ and NO generation in BAECs with repressed autophagy that were treated concurrently with exogenous ADP [5].

### PKC/PKD activation improves endothelial function in models of genetic and physiological autophagy compromise

Here we sought to discern the functional relevance of our strong in vitro evidence from BAECs and HAECs with autophagy compromise that targeting the link between P2Y1R stimulation and eNOS activation i.e., p-PKCδ^T505^ improves p-eNOS^S1177^ and NO generation. First, endothelial function was blunted in femoral and cerebral arteries from mice with genetic and physiological EC autophagy disruption, in a manner that could be recapitulated in arteries from the respective control mice upon eNOS enzyme inhibition. Importantly, in both models wherein EC autophagy was attenuated, arterial function could be reinstated by bryostatin- 1 treatment. Second, in light of our observation in vitro that bryostatin-1 activates downstream targets of p-PKCδ^T505^ (p-PKD^S744/S748^, p-PKD^S916^) to a greater extent than p-PKCδ^T505^ itself, and with evidence that the PKCδ inhibitor rottlerin exerts off-target effects [5], we reasoned that PKD inhibition might be the best approach to substantiate the functional importance of PKC/PKD signaling. Indeed, after verifying that bryostatin-1-induced p-PKD^S744/S748^, p-PKD^S916^, and p-eNOS^S1177^ was abrogated by concurrent CRT treatment in vitro, we likewise demonstrated that the ability for bryostatin-1 to rejuvenate intraluminal flow-mediated vasodilation in both models of EC autophagy compromise was lost by CRT treatment ex vivo. We conclude that bryostatin-1 activates PKC/PKD signaling in arteries from mice with blunted EC autophagy to improve endothelial function.

The bryostatins are a family of ~ 20 macrolide natural products isolated from the marine bryozoan *Bugula neritina *[36, 37]. The biologically active constituent bryostatin-1 crosses the blood brain barrier [38–40] and binds to the regulatory C1 domain of the PKC isoforms [41]. Clinical trials and pre-clinical investigations have documented the safety and therapeutic utility of bryostatin-1 in the context of neurodegenerative diseases and stroke [38, 39, 42–46]. For instance, preclinical models of Alzheimer's disease (AD) suggest bryostatin-1 enhances neuronal processes, and clinical studies of AD and MS patients indicate bryostatin-1 augments the formation and maintenance of synapses and/or beneficially modifies neuronal connections [46]. Regarding stroke, older rats challenged with occlusion of the middle cerebral artery for 2 h, followed by recombinant tissue plasminogen activator (r-tPA) -mediated reperfusion, displayed reduced infarct volume, greater neurological function, and improved survival rate if treated with bryostatin-1 vs. saline starting at 6 h reperfusion [38]. In another study from the same laboratory group, protection from ischemic brain injury was observed when bryostatin-1 vs. vehicle was administered immediately upon reperfusion, followed by r-tPA administration at 6 h [39]. While improved cerebral blood flow has potential to mitigate pathologies associated with both AD and cerebral ischemia, the impact of bryostatin-1 on vascular function has not been examined in either context to our knowledge. This is particularly important in the case of acute ischemic stroke because collateral blood flow during ischemia, and cerebral blood flow upon reperfusion, are important predictors of stroke outcome [47]. As proof of concept for future experiments, we show bryostatin-1 dilates parenchymal arterioles from older mice. Studies are ongoing to evaluate whether bryostatin-1 delivery during transient middle cerebral artery occlusion and/or upon reperfusion lessens the severity of tissue injury and neurobehavioral and motor deficits in response to acute ischemic stroke in older mice that may have a concurrent defect concerning EC autophagy.

## Supplementary Information

Below is the link to the electronic supplementary material.

Supplementary file1 (PDF 1730 KB)

Supplementary file2 (DOCX 21 KB)

## Data Availability

All data supporting the findings of this study are included in the main and supplementary materials. Further details are available from the corresponding author upon reasonable request.
