# Supplementary figures and images for: Bryostatin-1 improves function in arteries with suppressed endothelial cell autophagy
Source: GeroScience. 2025 Apr 12;48(1):351–66. doi: 10.1007/s11357-025-01650-5 (PMC12972466; doi:10.1007/s11357-025-01650-5)

Figure S1

A

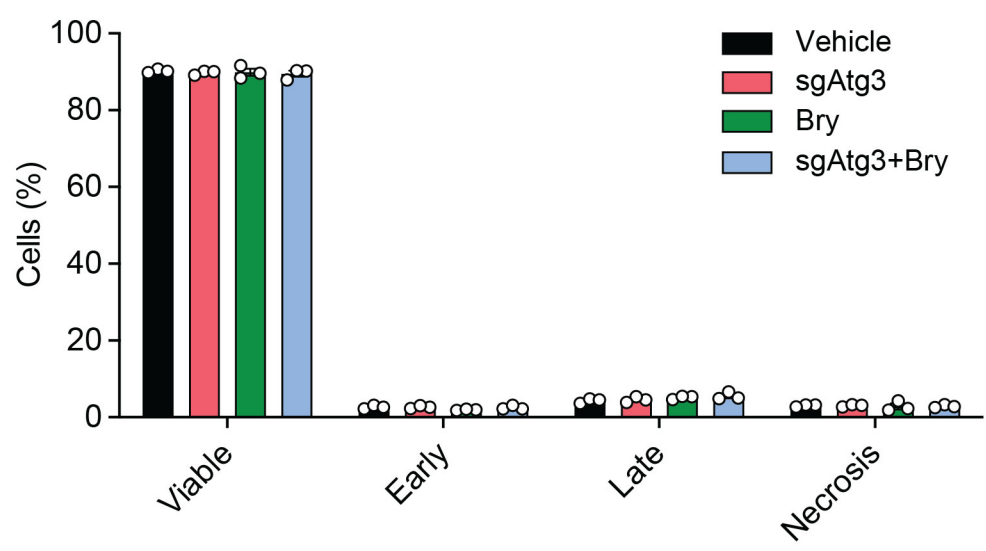

B

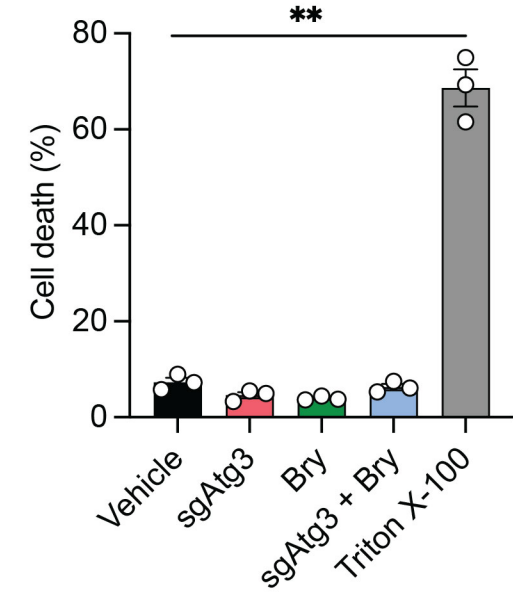

**Figure S2**

**A**

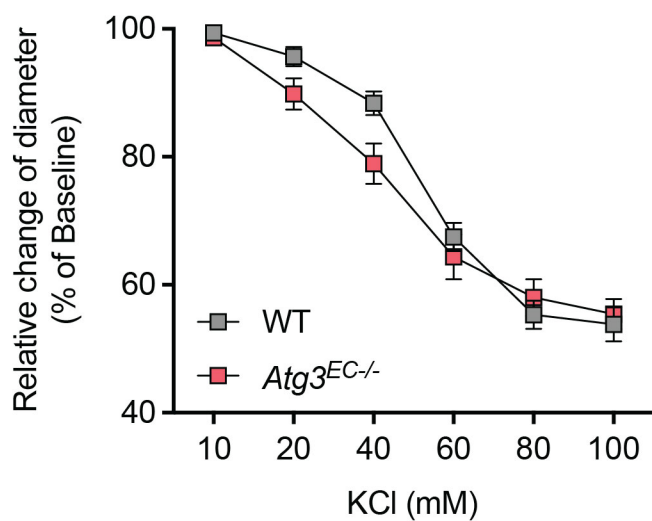

**B**

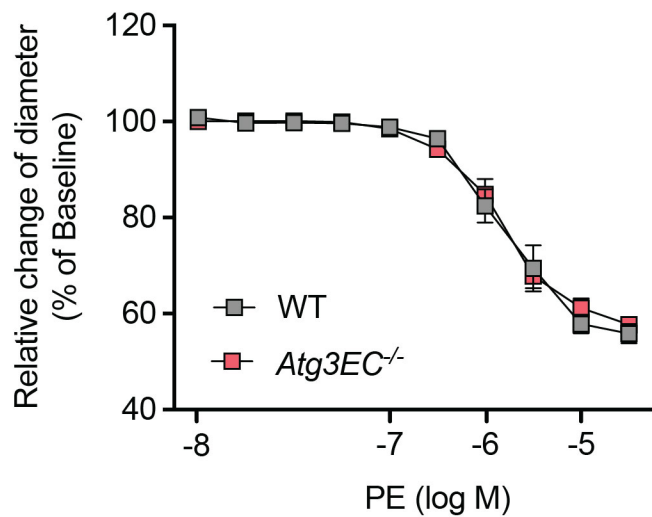

**C**

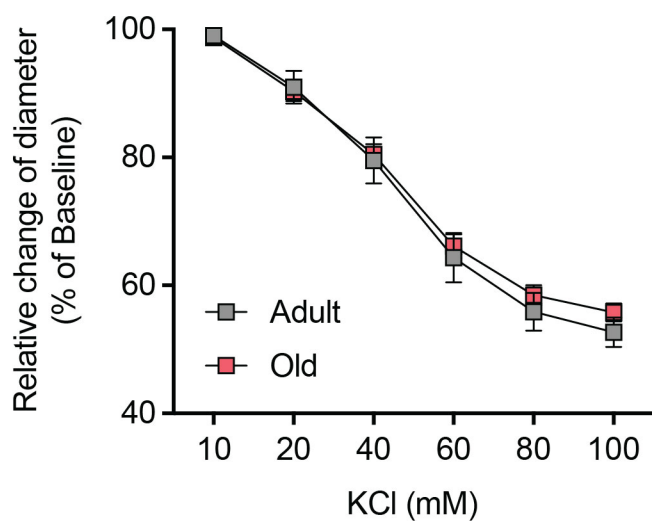

**D**

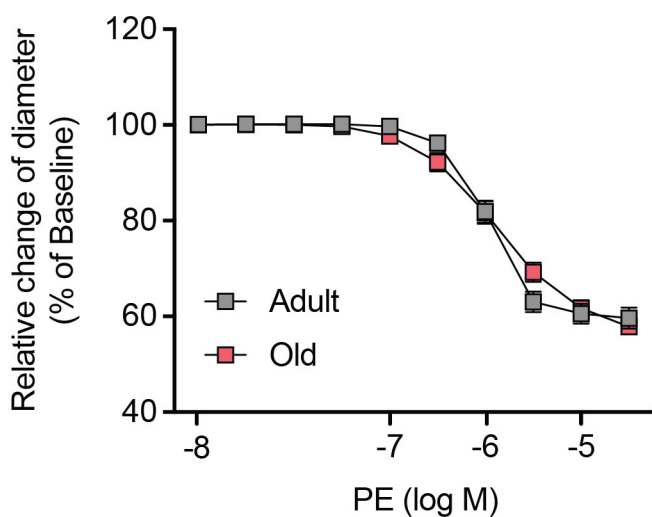

**Figure S3**

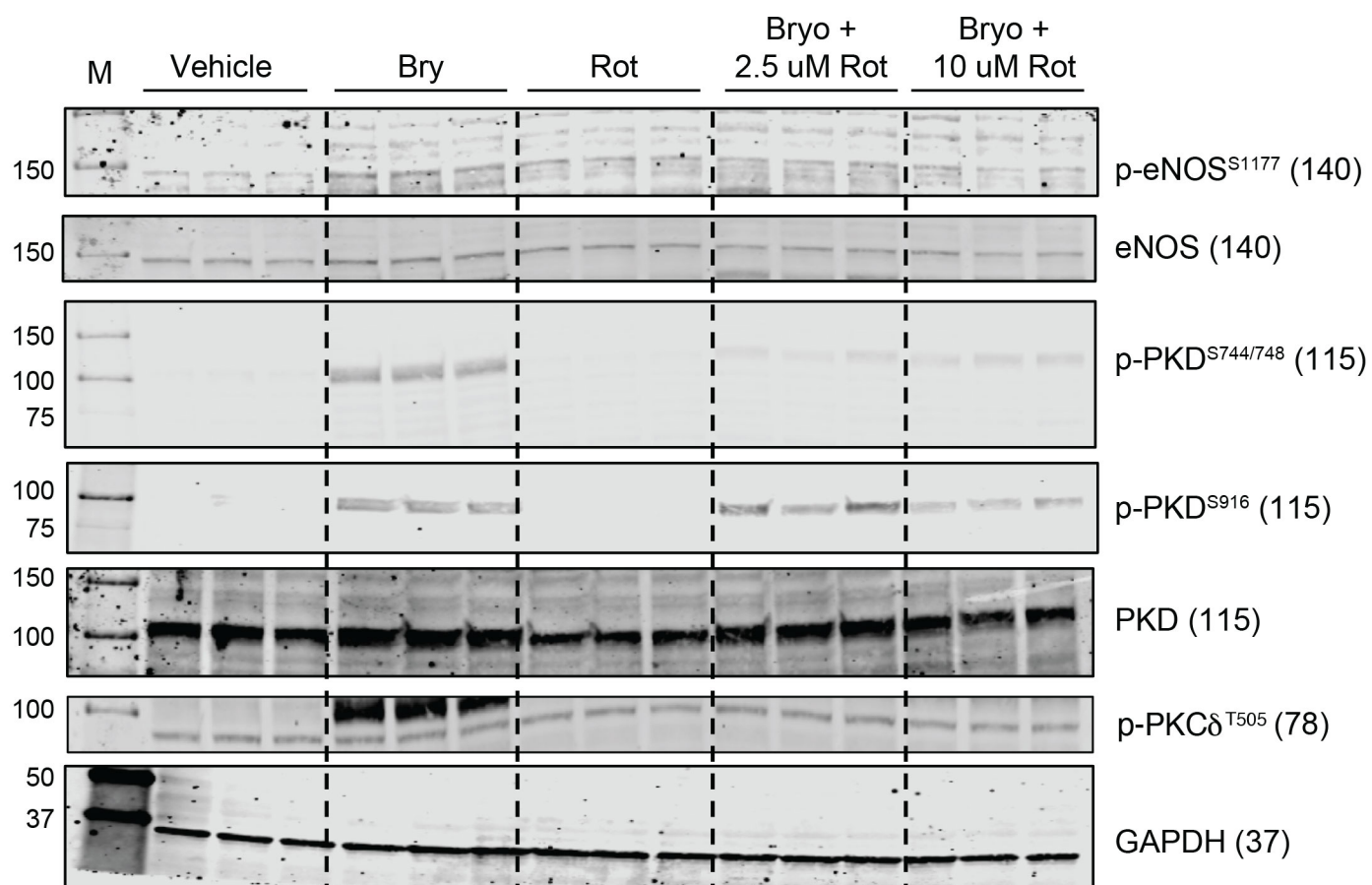

Figure S4

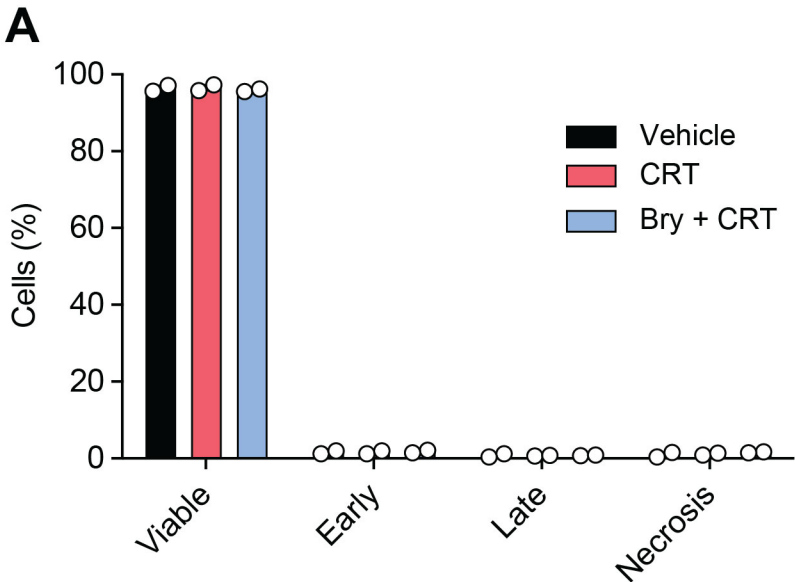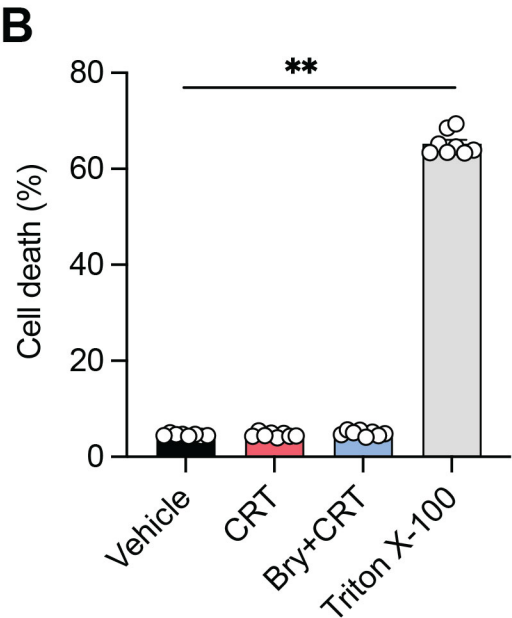

Supplement: Supplementary file 1 — Supplementary file1 (PDF 1730 KB) [file 11357_2025_1650_MOESM1_ESM.pdf]
