# Supplementary material for: Bryostatin-1 improves function in arteries with suppressed endothelial cell autophagy
Source: GeroScience. 2025 Apr 12;48(1):351–66. doi: 10.1007/s11357-025-01650-5 (PMC12972466; doi:10.1007/s11357-025-01650-5)
Supplement: Supplementary file 2 — Supplementary file2 (DOCX 21 KB) [file 11357_2025_1650_MOESM2_ESM.docx]

**Supplementary Table 1. Animal and vessel characteristics**

| ***Cohort1 (Figure 2)*** |  | | | |  | | | | | | |
| --- | --- | --- | --- | --- | --- | --- | --- | --- | --- | --- | --- |
|  | WT (n=4) | | | | *Atg3^EC-/-^* (n=4) | | | | | | |
| *Animal characteristics* | | | | | | | | | | | |
| Age, months | 3 | ± | 1 | | 3 | ± | | 1 | | | |
| Body weight, g | 27 | ± | 1 | | 28 | ± | | 1 | | | |
|  |  | | | |  | | | | | | |
| *Femoral a. characteristics* | | | | | | |  |  |  |  |  |
| Diameter at 0 mmHg, µm | 245 | ± | 11 | | 205 | ± | | 4* | | | |
| Diameter at 60 mmHg, µm | 331 | ± | 6 | | 325 | ± | | 7 | | | |
| Femoral a. numbers | n=4 | | | | n=4 | | | | | | |
| ***Cohort 2 (Figure 3)*** |  | | | |  | | | | | | |
|  | Adult (n=5) | | | | Old (n=5) | | | | | |  |
| *Animal characteristics* | | | | | | |  |  |  |  |  |
| Age, months | 7 | ± | 1 | | 23 | ± | | 1 | | |  |
| Body weight, g | 31 | ± | 1 | | 33 | ± | | 1 | | |  |
|  |  | | | |  | | | | | |  |
| *Femoral a. characteristics* | | | | | | |  |  |  |  |  |
| Diameter at 0 mmHg, µm | 231 | ± | 5 | | 248 | ± | | 8 | | |  |
| Diameter at 60 mmHg, µm | 341 | ± | 7 | | 334 | ± | | 10 | | |  |
| Femoral a. numbers | n=6 | | | | n=6 | | | | | |  |
| ***Cohort 3 (Figure 5A)*** |  | | | |  | | | | | |  |
|  | *Atg3^EC-/-^ (n=4)* | | | |  | | | | | |  |
| *Animal characteristics* | | | | |  |  |  |  |  |  |  |
| Age, months | 3 | ± | | 1 |  |  |  |  |  |  |  |
| Body weight, g | 27 | ± | | 1 |  |  |  |  |  |  |  |
|  | *Atg3^EC-/-^ + Bry + L-NMMA* | | | | *Atg3^EC-/-^ + Bry + CRT* | | | | | |  |
| *Femoral a. characteristics* | | | | | | |  |  |  |  |  |
| Diameter at 0 mmHg, µm | 325 | ± | | 8 | 322 | ± | | | 7 | |  |
| Diameter at 60 mmHg, µm | 476 | ± | | 3 | 473 | ± | | | 8 | |  |
| Femoral a. numbers | n=4 | | | | n=4 | | | | | |  |
| ***Cohort 4 (Figure 5B)*** |  | | | |  | | | | | |  |
|  | Old (n=6) | | | |  | | | | | |  |
| *Animal characteristics* | | | | |  |  |  |  |  |  |  |
| Age, months | 23 | ± | | 1 | 23 | ± | | | | 1 |  |
| Body weight, g | 33 | ± | | 1 | 33 | ± | | | | 1 |  |
|  | *Old + Bry +  L-NMMA* | | | | *Old + Bry +*  *CRT* | | | | | |  |
| *Femoral a. characteristics* | | | | | | |  |  |  |  |  |
| Diameter at 0 mmHg, µm | 343 | ± | | 14 | 332 | ± | | | | 24 |  |
| Diameter at 60 mmHg, µm | 482 | ± | | 30 | 484 | ± | | | | 29 |  |
| Femoral a. numbers | n=6 | | | | n=6 | | | | | |  |

Values are means ± SE. Diameter, external diameter. *p<0.05 vs. WT using an unpaired t-test.
